# Spinal cord perfusion pressure correlates with breathing function in patients with acute, cervical traumatic spinal cord injuries: an observational study

**DOI:** 10.1186/s13054-023-04643-y

**Published:** 2023-09-20

**Authors:** Ravindran Visagan, Ellaine Boseta, Argyro Zoumprouli, Marios C. Papadopoulos, Samira Saadoun

**Affiliations:** 1 Academic Neurosurgery Unit, St. George’s, University of London, Cranmer Terrace, London, SW17 0RE UK; 2 Neuro-anaesthesia and Neuro-intensive Care Unit, St. George’s Hospital, London, SW17 0QT UK

**Keywords:** Diaphragm, Intensive care, Intercostal muscle, Monitoring, Perfusion pressure, Spinal cord injury

## Abstract

**Objective:**

This study aims to determine the relationship between spinal cord perfusion pressure (SCPP) and breathing function in patients with acute cervical traumatic spinal cord injuries.

**Methods:**

We included 8 participants without cervical TSCI plus 13 patients with cervical traumatic spinal cord injuries, American Spinal Injury Association Impairment Scale grades A–C. In the TSCI patients, we monitored intraspinal pressure from the injury site for up to a week and computed the SCPP as mean arterial pressure minus intraspinal pressure. Breathing function was quantified by diaphragmatic electromyography using an EDI (electrical activity of the diaphragm) nasogastric tube as well as by ultrasound of the diaphragm and the intercostal muscles performed when sitting at 20°–30°.

**Results:**

We analysed 106 ultrasound examinations (total 1370 images/videos) and 198 EDI recordings in the patients with cervical traumatic spinal cord injuries. During quiet breathing, low SCPP (< 60 mmHg) was associated with reduced EDI-peak (measure of inspiratory effort) and EDI-min (measure of the tonic activity of the diaphragm), which increased and then plateaued at SCPP 60–100 mmHg. During quiet and deep breathing, the diaphragmatic thickening fraction (force of diaphragmatic contraction) plotted versus SCPP had an inverted-U relationship, with a peak at SCPP 80–90 mmHg. Diaphragmatic excursion (up and down movement of the diaphragm) during quiet breathing did not correlate with SCPP, but diaphragmatic excursion during deep breathing plotted versus SCPP had an inverse-U relationship with a peak at SCPP 80–90 mmHg. The thickening fraction of the intercostal muscles plotted versus SCPP also had inverted-U relationship, with normal intercostal function at SCPP 80–100 mmHg, but failure of the upper and middle intercostals to contract during inspiration (i.e. abdominal breathing) at SCPP < 80 or > 100 mmHg.

**Conclusions:**

After acute, cervical traumatic spinal cord injuries, breathing function depends on the SCPP. SCPP 80–90 mmHg correlates with optimum diaphragmatic and intercostal muscle function. Our findings raise the possibility that intervention to maintain SCPP in this range may accelerate ventilator liberation which may reduce stay in the neuro-intensive care unit.

**Supplementary Information:**

The online version contains supplementary material available at 10.1186/s13054-023-04643-y.

## Background

Traumatic spinal cord injury (TSCI) is a devastating event, with annual global age-standardised incidence rate estimated at 13 patients per 100,000 people in 2016 [1]. There are no neuro-intensive care unit (NICU) treatments proved to improve neurological outcome. To facilitate the management of acute TSCI, we developed monitoring from the injured cord. We insert a pressure probe intradurally at the injury site to record intraspinal pressure (ISP) [2], used to compute spinal cord perfusion pressure (SCPP), analogous to intracranial and cerebral perfusion pressures in traumatic brain injury. SCPP, the net pressure that drives blood flow to the injured cord, is a key physiological parameter in TSCI. Increasing SCPP reduces cord ischaemia (tissue glucose rises [3], tissue oxygen (p_sct_O_2_) rises [4], tissue lactate/pyruvate ratio (LPR) falls) [3], enhances cord autoregulation [5], and improves motor [6], sensory [7], urinary bladder [8], and anal sphincter [9] functions.

Cervical TSCI is more disabling than thoracic TSCI, because cervical TSCI weakens the upper limbs and impairs breathing, blood pressure and body temperature control. Cervical TSCI causes diaphragmatic (nerve root supply C3–C5), intercostal (nerve root supply T1–T11), and abdominal wall (T7–L1) muscle dysfunction, leading to reduced vital capacity from muscle fatigue and secretion retention from impaired coughing [10]. The consequences are pneumonia, failed extubation, tracheostomy, and prolonged NICU stay [11]. Therefore, intervention to enhance breathing in acute cervical TSCI in NICU would be a major advance.

Our study aimed to determine whether SCPP affects the function of breathing muscles in patients with acute, cervical TSCI. We used ultrasound scanning (USS) to monitor non-invasively changes in diaphragmatic and intercostal muscle thickness and diaphragmatic excursion during breathing. We also recorded the electrical activity of the diaphragm (EDI) with NAVA (neurally adjusted ventilatory assist) nasogastric tubes [12, 13].

## Methods

### Institutional research board approvals

TSCI patients were recruited as part of the Injured Spinal Cord Pressure Evaluation (ISCoPE) clinical study at St. George's Hospital, London, UK. Approvals for ISCoPE, including consent form and patient information sheet were obtained from the St. George's, University of London Joint Research Office, and the National Research Ethics Service London–St Giles Committee (No. 10/H0807/23). The study was performed in accordance with ethical standards laid down in the 1964 Declaration of Helsinki and its later amendments. Informed consent was obtained from all participants included in the study or their family members. ISCoPE is registered at www.clinicaltrials.gov and www.ichgcp.net as NCT02721615. Inclusion criteria were: (1) TSCI American Spinal Injury Association Impairment Scale (AIS) grade A, B, or C; (2) age 18–70 years; (3) timing between TSCI and surgery within 72 h. Exclusion criteria were: (1) patient unable to consent; (2) other significant co-morbidities; (3) penetrating TSCI.

### Participants

Data were collected from 21 participants. We included all 13 cervical TSCI patients recruited to the ISCoPE study between September 2020 and January 2023 with viable ISP, SCPP, microdialysis (MD), and p_sct_O_2_ monitoring signals. Recordings of breathing function were made from patients when not attached to a ventilator or, if attached to a ventilator, patients initiated their own breaths with pressure support (PS) and positive end-expiratory pressure (PEEP) as chosen by the NICU team. We also included 8 controls (5 healthy participants, 3 thoracolumbar spinal injuries) who consented to ultrasound examination to establish technique feasibility, reproducibility, and normal parameters in non-TSCI participants. AIS classifications in the 3 thoracolumbar injuries were T8A, T9A and L1C. Lower intercostal function in the 2 thoracic injuries were excluded from final analysis.

### Neurosurgical management

All patients were admitted to the neurosurgical unit at St. George's Hospital and underwent International Standards for Neurological Classification of Spinal Cord Injury (ISNCSCI) assessments by a trained neurosurgical resident, which was repeated at 2–3 weeks post-TSCI and 6-month follow-up. Surgical decompression and spinal instrumentation were performed by a neurosurgeon. The type of surgery was based on clinical indication and surgeon preference and included posterior approach with lateral mass and/or pedicle screw plus rod fixation (Stryker Oasys for cervical; Stryker UK, Newbury, Berkshire, England). Anterior instrumentation, if performed, involved anterior cervical discectomy, placement of Tritanium-C Cage and Aviator plate (Stryker UK, Newbury, Berkshire, England). All patients received antibiotics at induction (cefuroxime 1.5g intravenously if no penicillin allergy) and 48 h of vancomycin and gentamicin postoperatively according to body weight as standard hospital protocol. Pre-operative CT and MRI spine, then post-operative CT spine were performed, typically within 2 days, and post-operative MRI spine, typically within 1 week after probe removal.

### SCPP monitoring

**Fig. 1:**
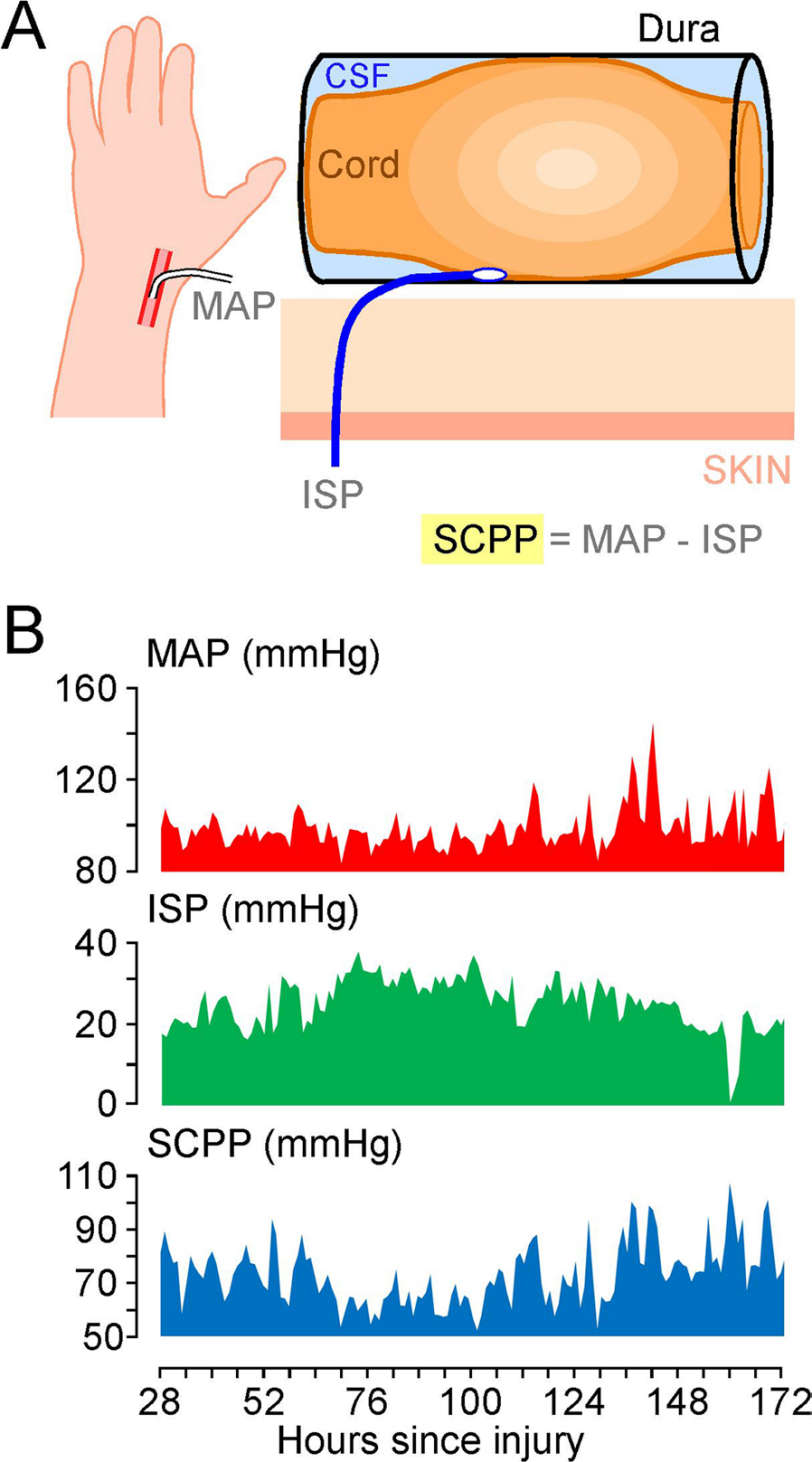
Monitoring from the injured spinal cord. **A** Pressure probe at the injury site monitors ISP, arterial line monitors MAP, and SCPP is computed as MAP–ISP. **B** Example of MAP, ISP and SCPP signals from a C4 AIS B TSCI patient

After posterior decompression of the spinal cord, under the operating microscope, a pressure probe (Codman Microsensor Transducer®, Depuy Synthes, Leeds, UK) was placed intradurally between the dura and injured cord at the site of maximal cord swelling and secured to the skin with silk sutures. The pressure probe was connected to a Codman ICP box. Arterial blood pressure (ABP) was recorded from a radial artery catheter. Both ISP and ABP interface with the ICU Philips Intellivue MX800 monitor (Philips, Guildford, U.K.), which is connected to a laptop running ICM+ recording software (Cambridge, U.K). ISP and ABP signals were sampled at 500 kHz and used to compute SCPP as mean arterial pressure (MAP) minus ISP. ISP is the same as intraparenchymal cord pressure at the injury site [14], which differs from cerebrospinal fluid (CSF) pressure measured above or below the injury because the swollen, injured cord is compressed against the dura, thus compartmentalising the intrathecal space as described in earlier publications [2, 15, 16]. Figure 1 shows the setup.

### NICU management

Postoperatively, all patients were admitted to the NICU and reviewed daily by NICU and neurosurgery teams. Each patient's ventilation was supported as required, with corresponding sedation and timing of post-operative extubation dependent on the level and severity of TSCI. This included either timely extubation postoperatively or early tracheostomy. The surgical wound was reviewed daily, a post-operative drain was kept on gravity for 1 week, and the patient was frequently turned in bed to avoid pressure sores. To prevent venous thromboembolism, we used Flowtrons® and started prophylactic low-molecular-weight heparin at 24 h. Vasopressor support (norepinephrine) was administered as follows: in general, MAP was kept at > 85 mmHg. If the MAP persistently fell < 85 mmHg (or rose > 100 mmHg), the norepinephrine dose was increased (or reduced) typically by 0.02 μg/kg/min, followed by assessment of the MAP response, prior to further titration to achieve the desired effect. Norepinephrine was not given to patients who self-maintained MAP > 85 mmHg. NICU physicians did not act on ISP, SCPP, p_sct_O_2_ or MD values in line with the study's observational nature. The ISP, MD and p_sct_O_2_ probes were removed within a week, and the probe skin exit sites were sutured using nylon.

### Electrical activity of the diaphragm

**Fig. 2:**
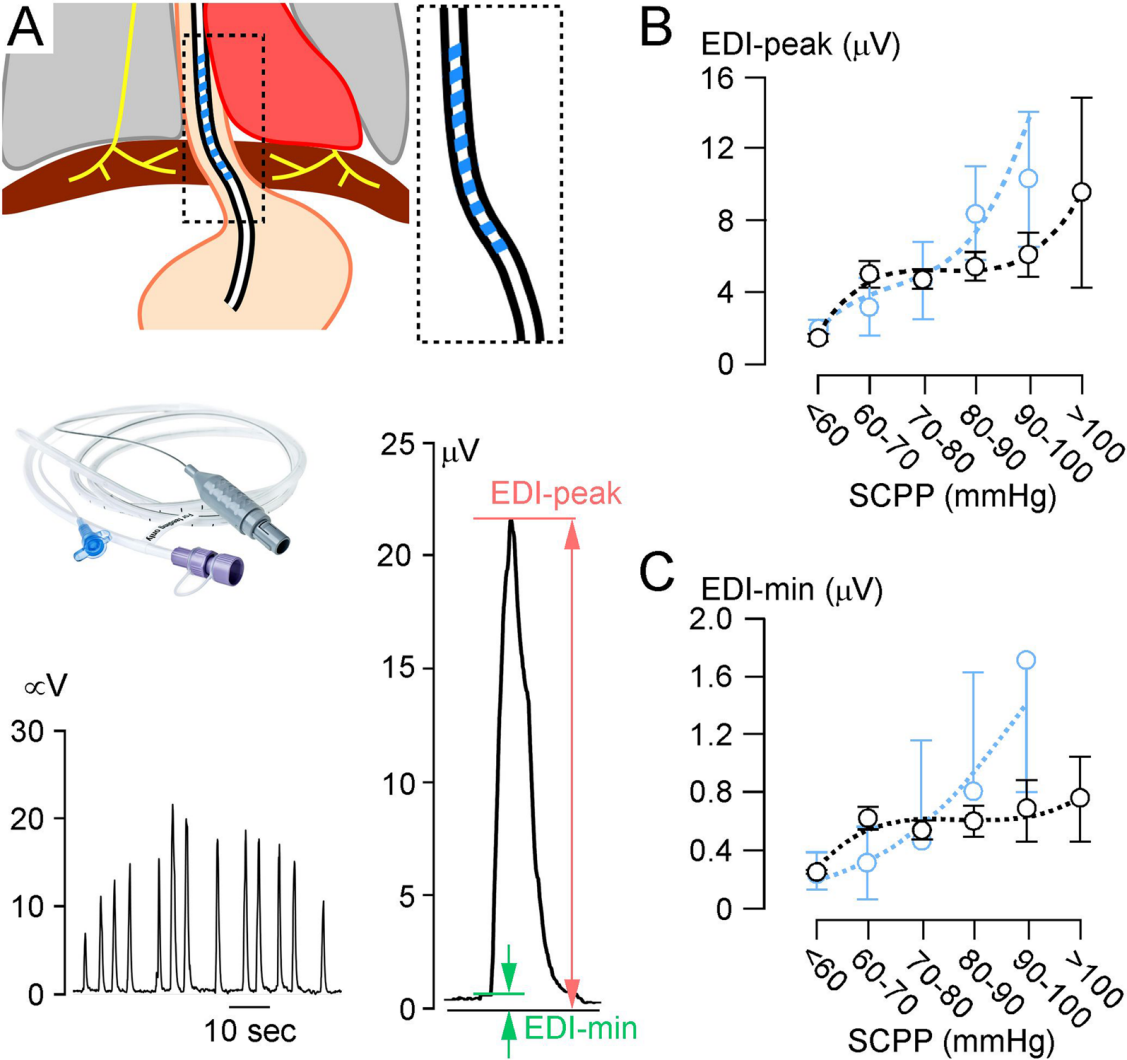
Electrical activity of the diaphragm (EDI). **A** (top left) Schematic for EDI monitoring. (top right) Magnified view of EDI nasogastric tube with electrode contacts (blue). (middle) Phot of an EDI nasogastric tube. (bottom left) EDI recordings from a C8 A TSCI patient. (bottom right) Magnified view of EDI signal showing EDI-min and EDI-peak. Diaphragm EMG versus SCPP for **B** EDI-peak, and **C** EDI-min. Black, all recordings; blue, recordings when not attached to ventilator. Mean ± standard error. Best-fit polynomial curves (**B** black, cubic: *r*^2^ = 0.90, **B** blue cubic: *r*^2^ = 0.98; **C** black, cubic: *r*^2^ = 0.98; **C** blue, quadratic: *r*^2^ = 0.95). Sample sizes in Additional file 8

EDI was captured by the modified NAVA nasogastric tube (MAQUET, Solna, Sweden) inserted by NICU staff for feeding and to monitor EDI. Optimal catheter position was verified by X-Ray and ventilator onscreen signal guidance during placement. The EDI catheter was connected to the SERVO-U ventilator (Getinge, Göteborg, Sweden) via a specific EDI module (Maquet, Solna, Sweden). The Philips Intellivue MX800 monitor (Philips, Guildford, U.K.) was configured to the EDI signal through a laptop with ICM+ recording software (Cambridge, U.K). Two parameters were used: EDI-peak (neural inspiratory effort responsible for the size and duration of the breath) and EDI-min (tonic activity of the diaphragm that prevents de-recruitment of alveoli in expiration). Figure 2A shows the setup.

### Ultrasound scanning

Excursion of the left and right hemi-diaphragms was assessed with a 3.5–5.0 MHz GE C1–5 probe set on M-mode via the anterior subcostal window. During inspiration, the diaphragm contracts and moves caudally towards the transducer, recorded as upward motion on the M-mode trace. Excursion is the maximum vertical height of the M-mode signal from full expiration to inspiratory peak [17]. Diaphragmatic and intercostal muscle thickening was assessed bilaterally with a 10–15 MHz probe set on B-mode. Diaphragmatic thickness was measured as described [18, 19] at the zone of apposition where the diaphragm is directly behind the inner aspect of the lower rib cage, with values obtained for quiet and deep breathing. For intercostal USS, the probe was oriented in the sagittal plane to record from the 2nd/3rd intercostals (upper intercostals), 5th/6th intercostals (middle intercostals), all measured in the mid-clavicular line, and from the 9th–11th intercostals (lower intercostals) measured in the anterior axillary line. Landmarks were marked on each participant's chest to enhance reproducibility of measurements. On each occasion, measurements were made for up to a minute, and the mean of each parameter (diaphragmatic excursion and thickness, intercostal thickness) was computed. Diaphragmatic or intercostal muscle thickening fraction during breathing was defined as: (thickness_max_ − thickness_min_)/thickness_min_ × 100. We used the average of corresponding left and right measurements for each patient. Our diaphragm USS method aligns with recent expert consensus [20] and international guidelines [21].

### Optimising assessments of breathing function

Following pilot studies in participants without cervical TSCI (not shown), we eliminated the effect of patient position on EDI and USS parameters by performing all measurements with the patients sitting at 20°–30° in bed. Patients did not have chest infection, confirmed on routine chest X-ray, during the measurements. USS and EDI recordings were done during spontaneous breathing to eliminate any confounding effects of mechanical ventilation. One investigator (RV) performed all USS recordings to eliminate inter-assessor variability. RV received training in respiratory USS from two consultant radiologists, who independently verified the method and measurement acquisition technique.

### Statistical analysis

SCPP was acquired as a continuous signal using ICM+. Each USS assessment took typically 0.5–1 h. We averaged the SCPP for the duration of each USS and EDI assessment. We used best-fit linear, quadratic, and cubic polynomials for breathing function versus SCPP monitoring data and computed coefficients of determination (*r*^2^) with data shown as mean ± standard error. The two norepinephrine groups (low vs high dose) were compared using two-tailed *t*-tests.

## Results

### Participant characteristics

We recruited 13 cervical TSCI patients. All participants and family members approached agreed for the patients to participate. Table 1 shows demographic information. Most (10/13) were males, and most (9/13) were younger than 60 years. On admission, 4/13 patients were AIS grade A, 7/13 grade B and 2/13 grade C. 10/13 patients had posterior surgery only, and 3/13 had combined anterior + posterior surgery. AIS grade at follow-up (on average 7.9 months) improved in 9, stayed the same in 3 and deteriorated in no one. 1/13 patient was not followed-up due to non-surgical mortality from chest sepsis after discharge. In 8 control participants (5 healthy + 3 thoracolumbar spinal injuries), we evaluated USS parameters in 38 exams. As in the TSCI group, most controls were male (7/8) and less than 60 years old (7/8), with mean age 41.1 years (range 27.0–63.9). No one had co-morbidities or chest injuries.

**Table 1:** Demographic characteristics of cervical TSCI patients

No.	Age (years)	Sex	AIS admission	AIS follow-up	Follow-up (months)	Hours ISP monitoring
1	23.0	M	C5A	C7B	12.04	151
2	35.0	M	C5A	C3A	6.90	158
3	24.0	M	C5C	C5D	10.01	122
4	21.0	M	C4B	C4B	6.25	160
5	61.0	F	C5B	Lost		93
6	62.0	M	C6B	C5C	6.85	159
7	50.4	M	C4B	C1D	8.05	106
8	71.4	F	C5B	C7D	10.55	116
9	37.0	M	C5B	C5B	8.71	113
10	54.3	F	C1C	C3D	7.72	98
11	20.1	M	C5B	C5C	6.25	56
12	70.6	M	C5A	C5C	6.51	14
13	44.5	M	C5A	C6B	4.74	112
					Total	1458

AIS, American Spinal Injury Association Impairment Scale; F, female; ISP, intra spinal pressure; M, male

### Patient complications

Table 2 lists complications in the cervical TSCI patient group. There was no post-operative CSF leak, wound infection, haematoma, or cord damage from the monitoring probes. 11/13 patients (84.6%) had non-compressive pseudomeningocele on the 2-week post-operative MRI, all conservatively managed. Non-surgical complications included chest infection in 7/13, urinary tract infection in 4/13 and delayed uncomplicated pressure ulcers successfully managed conservatively in 2/13.

**Table 2:** Complications of cervical TSCI patients

Complication	No. of patients	%
*Probe related/surgical*		
CSF leak	0	0.0
Wound infection	0	0.0
Meningitis	0	0.0
Cord damage from probes	0	0.0
Post-operative hematoma	0	0.0
Further surgery/return to theatre	0	0.0
Pseudomeningocele	11*	84.6
Post-traumatic syrinx	0	0.0
*Non-surgical/delayed*		
Chest infection	7	53.8
Urine infection	4	30.8
Line/blood infection	1	7.7
Pulmonary embolus or DVT	0	0.0
Pressure ulcer	2	15.4
Delirium in NICU	1	7.7
Mortality	1**	7.7

*All non-compressive on post-operative MRI. 3/11 total resolution on 6 months MRI

**1/11 non-surgical mortality from pulmonary sepsis several months after surgery

### Reproducibility and confounding factors

Based on 8 participants without cervical TSCI, the coefficients of variation for the USS measurements are: diaphragmatic excursion 7.5%, diaphragmatic thickening 5.3% (right 4.7%, left 5.8%), and intercostal thickening 3.0% (upper 3.7%, middle 2.0%, lower 3.3%). The TSCI patients received norepinephrine infusion during 81.6% of the USS and EDI measurements. To determine the effect of norepinephrine, we compared USS and EDI measurements made with TSCI patients on high (> 0.1 μg/kg/min) versus low (≤ 0.1 μg/kg/min) norepinephrine dose. High norepinephrine dose was associated with higher MAP (96.2 vs 93.0 mmHg, *P* < 0.005), higher ISP (16.9 vs 8.9 mmHg, *P* < 0.001), lower SCPP (79.9 vs 85.1 mmHg, *P* < 0.0005) and lower % muscle thickening change during breathing for lower intercostals (− 0.1 vs 4.6%, *P* < 0.05) with no effect on EDI-peak, EDI-min, diaphragmatic excursion (quiet + deep breathing), % diaphragmatic thickening change (quiet + deep breathing) or % muscle thickening change for upper and middle intercostals. EDI and USS recordings were not performed in patients with cardiorespiratory instability, significant pre-existing lung disease, pulmonary contusions, pneumonia, or pulmonary embolus that may have influenced diaphragmatic or intercostal muscle function. PS support and PEEP may influence the USS and EDI parameters; therefore, in addition to analysing all breathing function recordings, we also analysed the subgroup related to non-ventilated patients.

### SCPP correlates with injury site metabolism

We first determined whether SCPP correlates with injury site metabolism assessed using spinal cord tissue oxygen (p_sct_O_2_) and microdialysis (MD) monitoring from the injury site. Details of the monitoring techniques are in Additional file 1. The relations between p_sct_O_2_ versus SCPP and tissue glucose versus SCPP are inverted-U shaped, with the maximum at SCPP 70–90 mmHg, whereas increasing SCPP is associated with decreasing LPR (Additional file 2). These findings suggest that low SCPP indicates ischaemia and that both hypo- and hyper- perfusion may be detrimental to the injured spinal cord, with optimum SCPP at 70–90 mmHg.

### SCPP correlates with diaphragmatic function

**Fig. 3:**
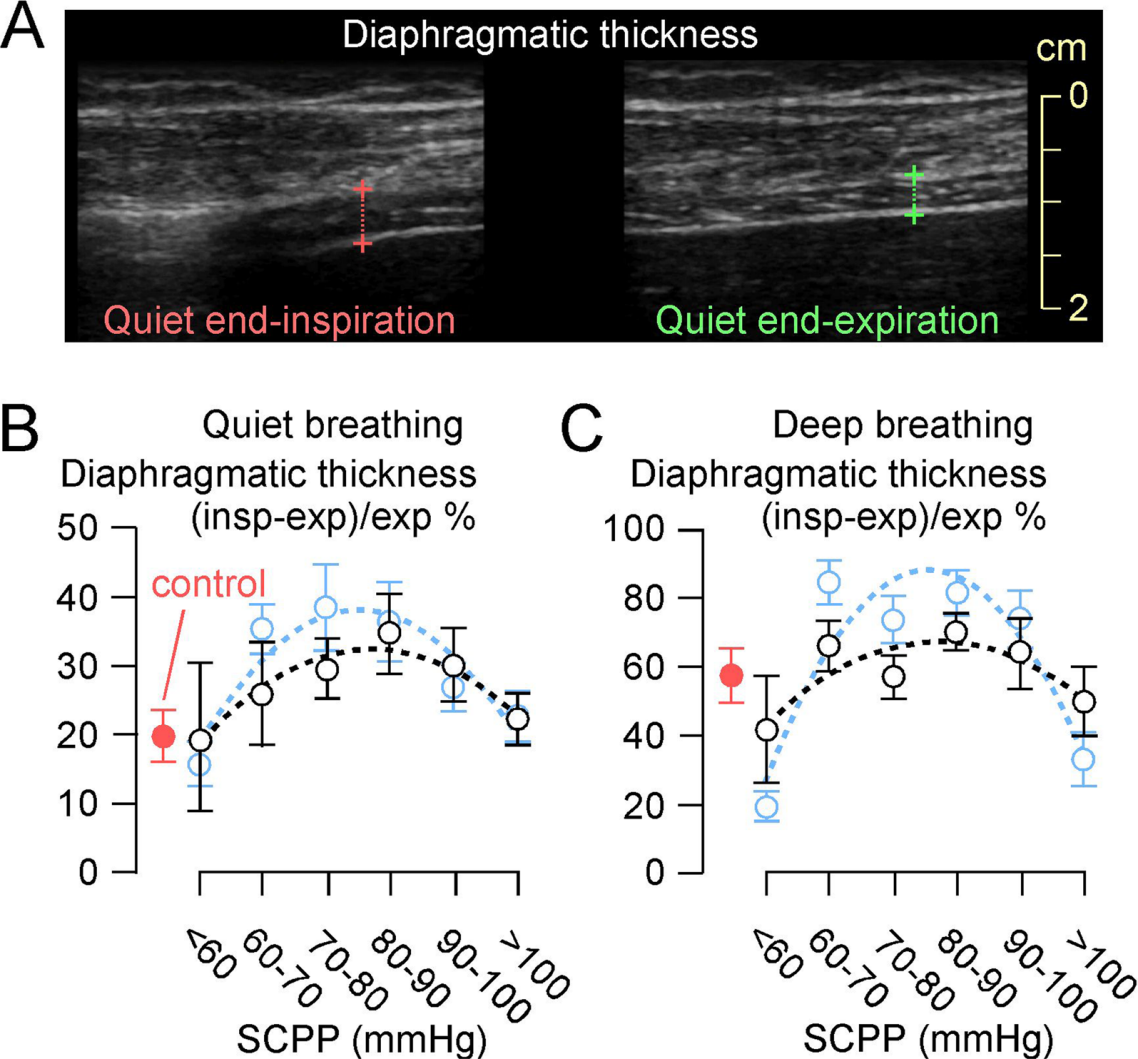
Diaphragmatic thickening fraction. **A** Photos of ultrasound exam of right hemi-diaphragmatic muscle thickening during quiet breathing, at end-inspiration and at end-expiration. % change in diaphragm thickness = (inspiration − expiration)/expiration plotted versus SCPP **B** during quiet breathing, and **C** during deep breathing. Black, all recordings; blue, recordings when not attached to ventilator. Red is data from controls without cervical TSCI. Mean ± standard error. Best fit quadratic (**B** black: *r*^2^ = 0.92; **B** blue: *r*^2^ = 0.85; **C** black: *r*^2^ = 0.72; **C** blue: *r*^2^ = 0.84). Sample sizes in Additional file 8

**Fig. 4:**
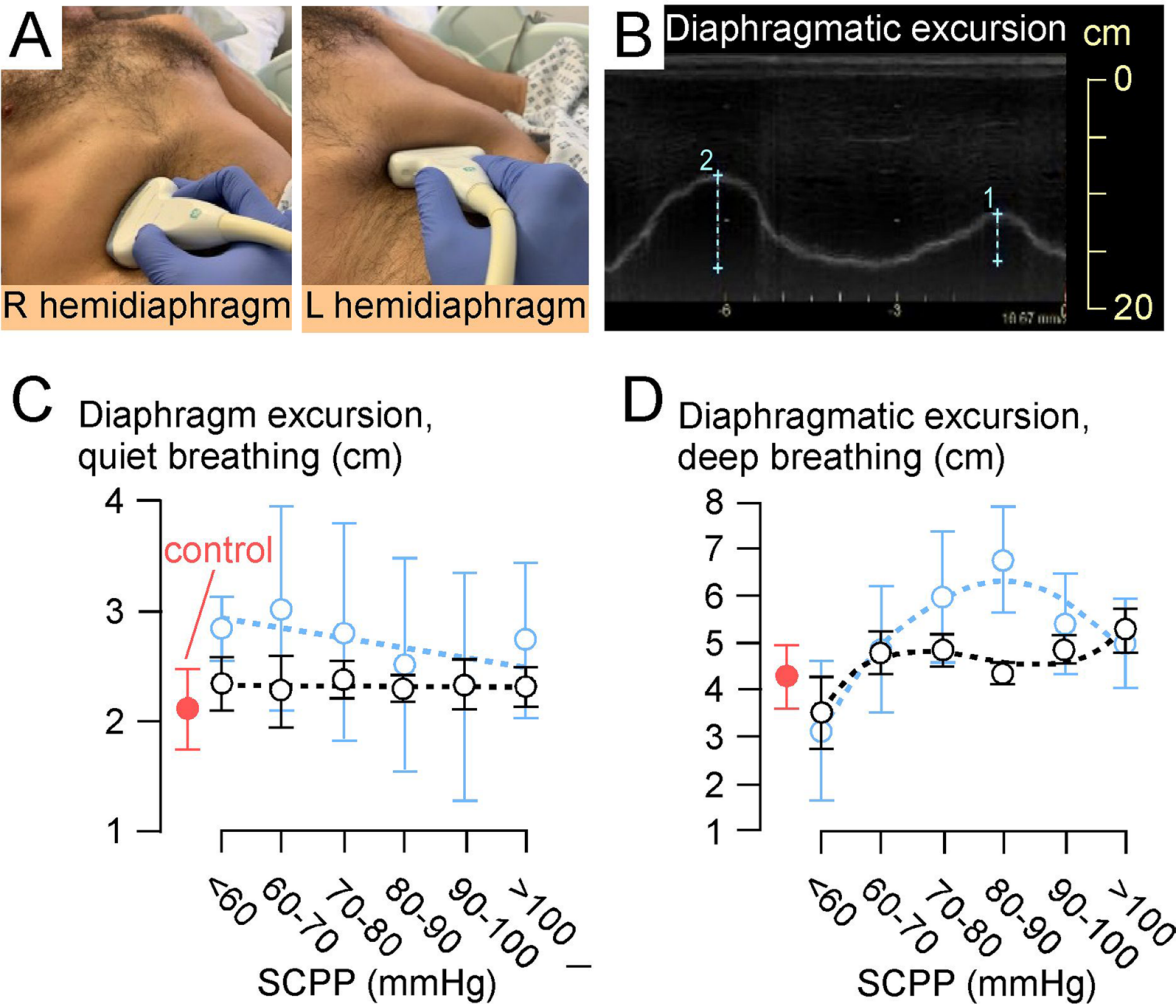
Diaphragmatic excursion. **A** Placement of ultrasound probe to record diaphragmatic excursion from (left) right hemidiaphragm, and (right) left hemidiaphragm. **B** Diaphragmatic excursion measurements in M-mode. Diaphragm excursion plotted versus SCPP **C** during quiet breathing, and **D** during deep breathing. Black, all recordings; blue, recordings when not attached to ventilator. Red is data from controls without cervical TSCI. Mean ± standard error. Best fit curve (**C** black, linear *r*^2^ = 0.00; **C** blue, linear *r*^2^ = 0.38; **D** black, cubic *r*^2^ = 0.79; **D** blue, quadratic *r*^2^ = 0.93). Sample sizes in Additional file 8

Figure 2B and C show the relationship between SCPP and EDI. SCPP < 60 mmHg is associated with low EDI-peak (diaphragmatic inspiratory effort) and EDI-min (diaphragmatic tonic activity). EDI-peak and EDI-min increase as SCPP increases > 60 mmHg, plateau at SCPP 60–90 mmHg, and increase further at SCPP > 90 mmHg. In non-ventilated patients, EDI varies with SCPP in a similar fashion. The diaphragmatic thickening fraction for quiet and deep breathing plotted versus SCPP has inverted-U relationship, with maxima at SCPP 80–90 mmHg (Fig. 3). Additional file 3 shows videos of normal and weak diaphragmatic contraction during quiet inspiration. During quiet breathing, there is no correlation between SCPP and diaphragmatic excursion, but during deep breathing, diaphragmatic excursion is low at SCPP < 60 mmHg, increases as SCPP increases > 60 mmHg, plateaus at SCPP 60–100 mmHg, and increases further at SCPP > 100 mmHg (Fig. 4). Diaphragmatic excursion when PEEP = 0 and PS = 0 also does not correlate with SCPP for quiet breathing but has inverse-U relationship with SCPP for deep breathing with peak at SCPP 80–90 mmHg. Additional file 4 shows videos of normal and reduced diaphragmatic excursion during deep inspiration. Figures 3 and 4 also show that the diaphragmatic thickening fractions and the diaphragmatic excursions for quiet and deep breathing in the control participants are generally low compared with the corresponding parameters in patients with cervical TSCI.

### SCPP correlates with intercostal muscle function

**Fig. 5:**
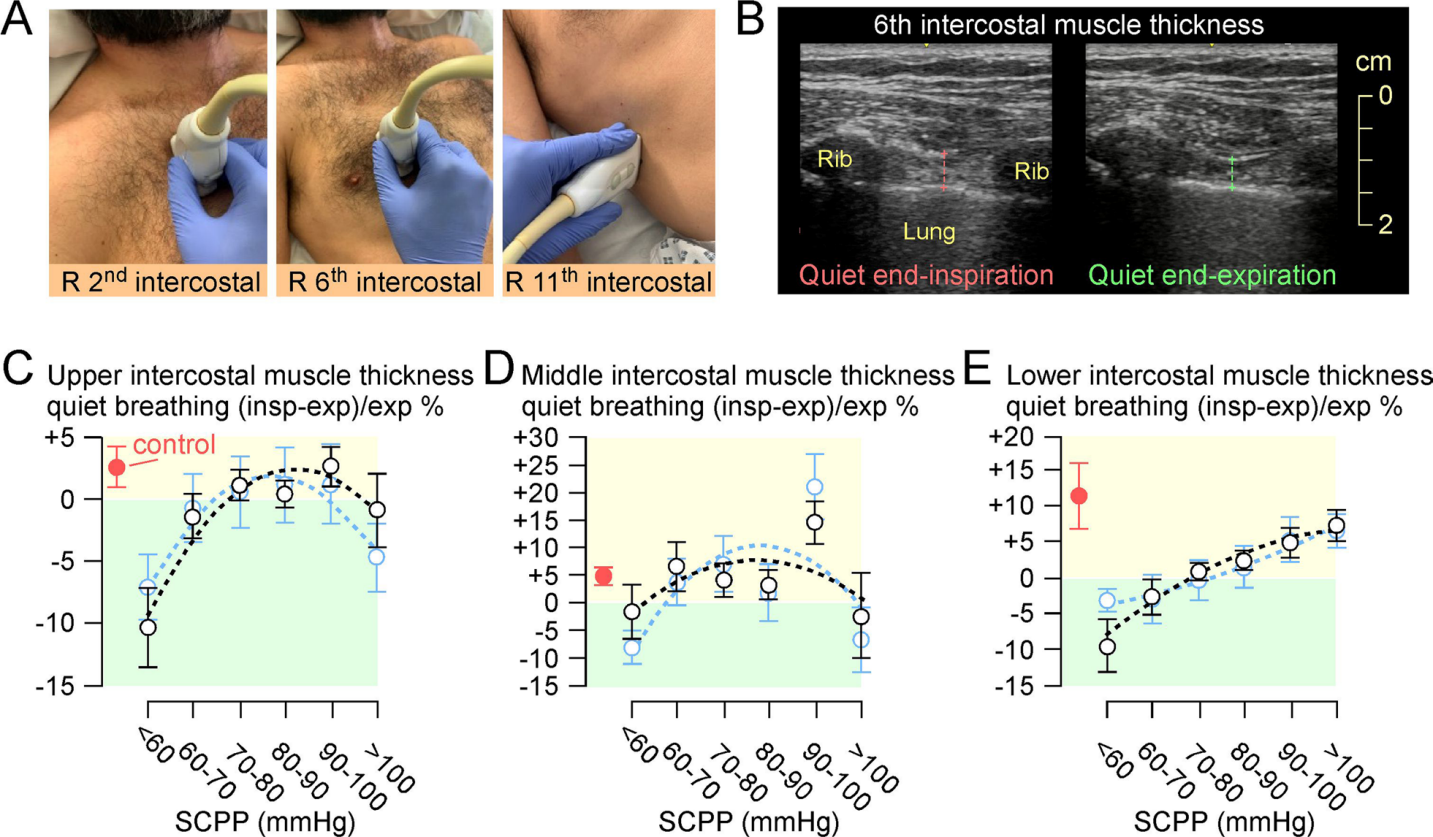
Intercostal muscles thickening fraction. **A** Placement of ultrasound probe to record excursion from (left) right 2nd intercostal muscle, (middle) right 6th intercostal muscle and (right) right 11th intercostal muscle. Photos of ultrasound exam of right 6th intercostal muscle thickening during quiet breathing, at end-inspiration and at end-expiration. % change in intercostal muscle thickness during quiet breathing = (inspiration − expiration)/expiration **B** in upper intercostal muscles, **C** in middle intercostal muscles, and **D** in lower intercostal muscles. Black, all recordings; blue, recordings when not attached to ventilator. Red is data from controls without cervical TSCI. Mean ± standard error. Best fit quadratic (**B** black: *r*^2^ = 0.91; **B** blue: *r*^2^ = 0.94; **C** black: *r*^2^ = 0.35; **C** blue: *r*^2^ = 0.45; **D** black: *r*^2^ = 0.98; **D** blue: *r*^*2*^ = 0.97). Sample sizes in Additional file 8

Figure 5 shows the relationship between the thickening fractions of the upper, middle, and lower intercostal muscles plotted against SCPP. SCPP < 70 mmHg (upper and lower intercostals) and < 60 mmHg (middle intercostals) was associated with negative intercostal thickening fraction, suggesting failure of the muscles to contract during inspiration (likely passive stretching, diaphragmatic breathing). For upper and middle intercostals, SCPP > 100 mmHg was also associated with failure of the muscles to contract during inspiration. Overall, SCPP 80–100 mmHg was optimal for allowing upper, middle, and lower intercostal muscles to contract during inspiration. Intercostal thickening fractions in non-ventilated patients correlate with SCPP in a similar fashion. Figure 5 also shows that, in the control participants, the upper, middle, and lower intercostals have positive thickening fractions, indicating intercostal muscle contraction during inspiration. Additional file 5 shows videos of normal and reduced intercostal muscle relaxation during quiet expiration.

### Individual patient data

Figures 2, 3, 4 and 5 combine data from all patients. We asked whether the observed correlations between the different measures of breathing function and SCPP are maintained for individual patients. The table in Additional file 6 summarises the correlations for each breathing function measure versus SCPP for each patient. Plots of all individual patient relationships are summarised in Additional file 7. This analysis shows that the correlations between breathing function and SCPP seen with combined patient data generally also hold for individual patients. However, some patients have no such correlations and, in the patients that have correlations, the SCPP that optimises breathing function varies between patients.

## Discussion

We showed that monitoring breathing function in critically ill patients with acute TSCI in NICU using USS and EDI is feasible and reproducible. Our key finding is that SCPP correlates with breathing: the data suggest that too low SCPP (cord hypo-perfusion) and too high SCPP (cord hyper-perfusion) are associated with more impaired breathing, whereas SCPP 80–90 mmHg correlates with improved diaphragmatic and intercostal muscle contractility. These findings still hold even when only data from non-ventilated patients are considered to eliminate the effects of PEEP and PS on the function of breathing muscles.

USS is a non-invasive, non-ionising technique that provides high-resolution images in real-time with good reproducibility [22]. Disadvantages include the confounding effects of inotropic support, PEEP and PS on the measurements, whereas obesity, reduced compliance from critically ill patients, and technical factors (such as patient position, probe location and angulation) also affect the recordings [23]. Subcostally scanning left hemi-diaphragmatic excursion is challenging because of the small splenic window and lung masking; we had failure rate of 70.9%, compared with subcostally scanning right hemi-diaphragmatic excursion. In the intensive care setting, diaphragmatic USS has been used to guide weaning from mechanical ventilation [17], evaluate diaphragmatic paralysis [24], and quantify the work of breathing [18]. We recommend devoting time to optimise the technique before using the data for clinical decision making.

The key diaphragmatic USS parameters are thickening fraction and excursion. Thickening fraction is a measure of the force of contraction of the diaphragm during inspiration [25]. Excursion is the diaphragm's up-and-down motion that contributes to the inspiratory lung volume [26]. Because of impaired intercostal muscle function, patients with cervical TSCI rely almost exclusively on diaphragmatic contraction to increase lung volume during inspiration. By comparison, in normal participants, diaphragmatic contraction is responsible for 70% of the tidal volume and the intercostal muscles for 30% [27]. This may explain our observation that diaphragmatic thickening fraction and excursion are generally higher in patients with cervical TSCI than in control participants with intact intercostal muscle function. Our data show that, during quiet (tidal) breathing in patients with cervical TSCI, diaphragmatic excursion is independent of SCPP (i.e. the tidal volume is reached regardless of the SCPP). However, SCPP affects the force of diaphragmatic contraction, measured using the thickening fraction, with the strongest contraction at SCPP 80–90 mmHg. Deep breathing requires increased diaphragmatic effort to inflate and deflate the lungs to vital capacity; we found that, during deep breathing, SCPP has a major influence on diaphragmatic thickening fraction and excursion with optimum function at SCPP 80–90 mmHg.

Diaphragmatic function was further assessed using the EDI nasogastric tube, initially designed for NAVA ventilation [12, 13]. During quiet breathing, normal EDI-peak is 5–10 μV, and normal EDI-min is up to 1 μV. EDI-peak increases when the diaphragm has to contract more, e.g. during respiratory distress, the EDI-peak may increase to 5–7 times above normal [28, 29]. Our data show that the EDI-peak is too low at SCPP < 60 mmHg, and that SCPP 60–100 mmHg is required for EDI-peak to normalise. We also found that low SCPP (< 60 mmHg) is associated with reduced EDI-min, a marker diaphragmatic tone marker. Reduced diaphragmatic tone is detrimental by allowing the abdominal contents to displace the diaphragm cranially, thus causing lung compression and atelectasis [30]. We found that SCPP 60–100 mmHg increases EDI-min, which may, in turn, facilitate tonic diaphragmatic contraction.

We also assessed the thickening of intercostal muscles, which are easily accessible by USS. Normally, the contribution of intercostal muscles to breathing increases with intense respiratory effort [31]. In our control group, the thickening fraction was low (≤ 5% for upper and middle intercostal muscles, 10% for low intercostals). In the cervical TSCI patients, we found an inverted-U relationship between intercostal thickening fraction and SCPP for upper and middle intercostals, with the maximum intercostal thickening fraction reaching normal level during quiet breathing at SCPP 80–90 mmHg. The thickening fraction of low intercostal muscles increased with increasing SCPP. Negative thickening fraction, seen during spinal cord hypo- and hyper-perfusion, likely indicates passive stretching of the intercostal muscles during inspiration that may occur with diaphragmatic (abdominal) breathing.

Plots of individual patient breathing function versus SCPP, rather than using data from all patients combined, raises some interesting issues. First, the inverted U relationship between breathing function and SCPP, observed with the combined patient data, also seems to hold for individual patients in most cases. Second, the SCPP that optimises breathing function may vary between patients. Third, in some patients, breathing function is very sensitive to change in SCPP, but in others less so, ie. the gradient of the inverted-U relationship varies between patients. Finally, in some patients, no inverted-U correlation between breathing function and SCPP was detected; this could be because an optimum SCPP does not exist or because the optimum SCPP lies outside the range 60–100 mmHg. We urge caution when interpreting data from individual patients due to the small sample size.

A limitation of our study is the small number of patients. This precludes multivariate analysis to determine the effect of factors such as patient age, severity of injury, and level of injury on the relationship between breathing function and SCPP. However, the conclusions for the combined patient data are supported by 1458 h of SCPP monitoring and 106 serial USS examinations (total 1370 images/videos). The NICU setting with critically ill patients required to cooperate during the assessments was particularly challenging. Our findings raise the possibility that intervention to maintain SCPP 80–90 mmHg may improve breathing in patients with acute, cervical TSCI. A future refinement would be analysis of single patient data to allow individualised management, rather than applying the optimum SCPP obtained from combined patient data to all patients. This would require modification of the USS technique to provide more datapoints per patient for real-time computation of the SCPP that optimises breathing function.

## Conclusions

Acute cervical TSCI impairs breathing. Our study shows that SCPP 80–90 mmHg correlates with improved breathing function. Further studies are required to determine whether SCPP-driven protocol accelerates ventilator liberation and reduces NICU length of stay.

### Supplementary Information

**Additional file 1**: Supplementary methods

**Additional file 2**: Relations between SCPP and monitored parameters

**Additional file 3**: Diaphragmatic ultrasound videos, B-mode

**Additional file 4**: Diaphragmatic excursion videos, M-mode

**Additional file 5**: Intercostal muscle ultrasound videos

**Additional file 6**: Summary of correlations between individual patient’s breathing function vs SCPP

**Additional file 7**: Plots of relations between individual patient’s breathing function vs SCPP

**Additional file 8**: Sample sizes for each graph

## Data Availability

The datasets used and/or analysed during the current study are available from the corresponding author on reasonable request.

## Abbreviations

ABP: Arterial blood pressure
AIS: American Spinal Injury Association Impairment Scale
EDI: Electrical activity of the diaphragm
ISP: Intraspinal pressure
LPR: Lactate/pyruvate ratio
MAP: Mean arterial pressure
MD: Microdialysis
NAVA: Neurally adjusted ventilatory assist
NICU: Neuro-intensive care unit
p_sct_O_2_: Partial pressure of spinal cord tissue oxygen
SCPP: Spinal cord perfusion pressure
TSCI: Traumatic spinal cord injury
USS: Ultrasound scan
