# Supplementary material for: Spinal cord perfusion pressure correlates with breathing function in patients with acute, cervical traumatic spinal cord injuries: an observational study
Source: Crit Care. 2023 Sep 20;27:362. doi: 10.1186/s13054-023-04643-y (PMC10512582; doi:10.1186/s13054-023-04643-y)
Supplement: Supplementary file 1 — Additional file 1: Supplementary methods [file 13054_2023_4643_MOESM1_ESM.pdf]

ADDITIONAL FILE 1 – Supplementary methods

Tissue oxygen monitoring. An O_2 electrode (Licox - CC1P1, Integra, Sophia-Antipolis, France) was inserted intradurally at the site of maximal spinal cord swelling and was connected to a Licox P_tO_2 Monitor (Integra, Integra, Sophia-Antipolis, France), linked to a Philips Intellivue MX800 monitor (Philips, Guildford, UK), and connected to the laptop running ICM+ (Cambridge, U.K.). The Licox signal (Fig. 1) was sampled at 500 kHz.

MD monitoring. A CMA61 MD catheter (CMA microdialysis AB, Solna, Sweden) was inserted intradurally at the site of maximal spinal cord swelling. MD was started postoperatively in the NICU as described[19–21]. Central nervous system fluid (CMA microdialysis AB) was perfused at 0.3 µL/min using the CMA106 pump (CMA microdialysis AB). MD vials were changed hourly and analysed using ISCUS Flex (CMA microdialysis AB) for glucose, lactate, and pyruvate. LPR was calculated. The first two samples from each patient were discarded to allow priming of the MD catheter and stabilisation of the metabolite concentrations. 100-fold changes in metabolite concentration, compared with the preceding hour, were excluded from analysis. Our MD method measures cord surface metabolism at the injury site, which correlates with intraparenchymal injury site metabolism, but differs from metabolites measured from lumbar CSF[19–21].
