# Supplementary material for: Spinal cord perfusion pressure correlates with breathing function in patients with acute, cervical traumatic spinal cord injuries: an observational study
Source: Crit Care. 2023 Sep 20;27:362. doi: 10.1186/s13054-023-04643-y (PMC10512582; doi:10.1186/s13054-023-04643-y)
Supplement: Supplementary file 2 — Additional file 2: Relations between SCPP and monitored parameters [file 13054_2023_4643_MOESM2_ESM.pdf]

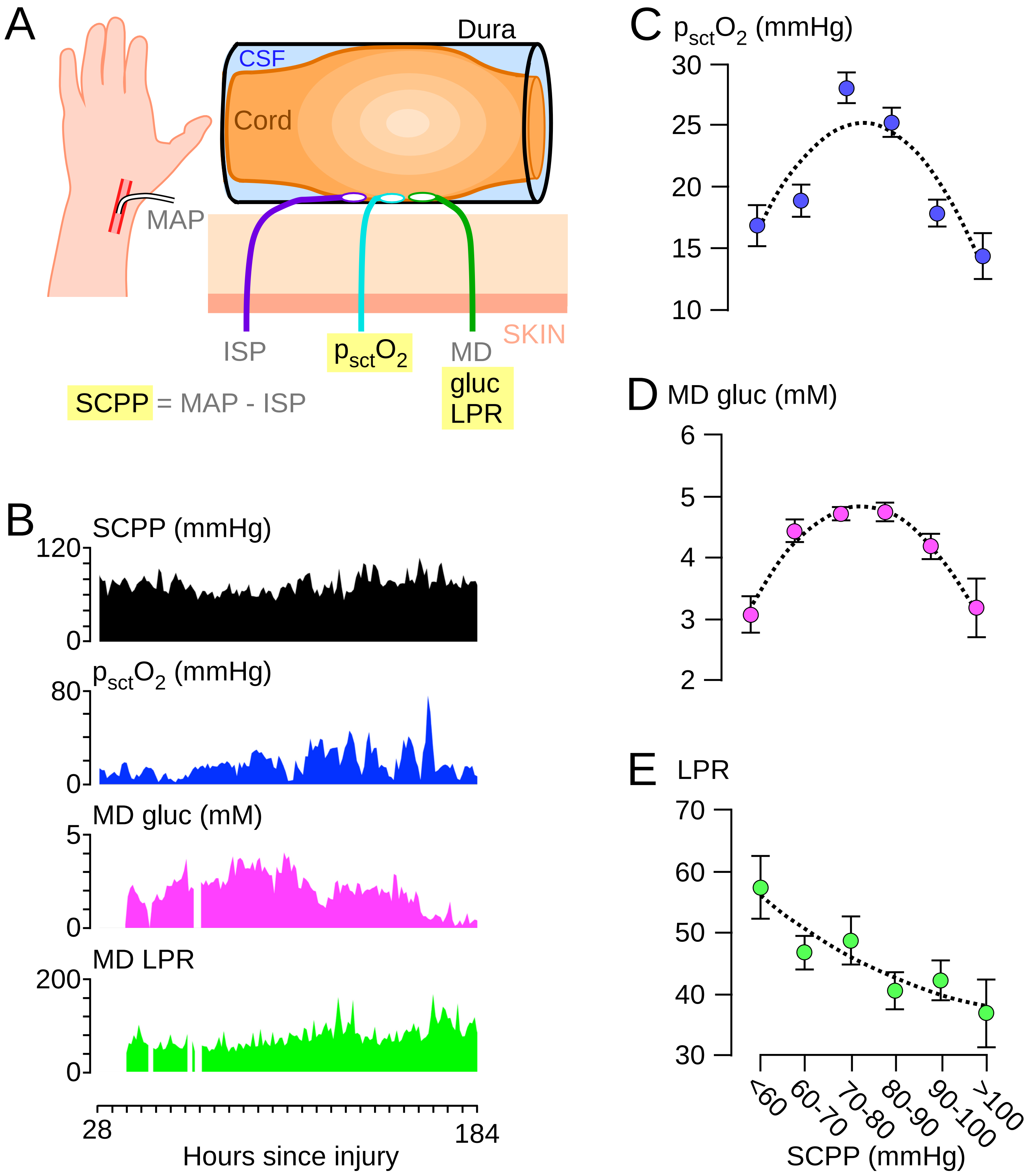

ADDITIONAL FILE 2. Monitoring setup. A. Monitoring from the injured spinal cord of Mean Arterial Pressure (MAP), Intra Spinal Pressure (ISP), Spinal Cord Perfusion Pressure (SCPP), Micro Dialysis (MD) to detect tissue glucose and tissue Lactate to Pyruvate Ratio (LPR), as well as tissue oxygen ($p_{sct}O_2$). **B.** Example of multimodality monitoring from a patient with cervical TSCI (21 years old, C4 ASIA B. Plots of **C.** $p_{sct}O_2$ *vs.* SCPP, **D.** tissue glucose *vs.* SCPP, and **E.** tissue LPR *vs.* SCPP. Mean $\pm$ standard error. Best-fit quadratics (D: $r^2 = 0.78$, E: $r^2 = 0.98$, F: $r^2 = 0.87$).
