# Supplementary figures and images for: Spinal cord perfusion pressure correlates with breathing function in patients with acute, cervical traumatic spinal cord injuries: an observational study
Source: Crit Care. 2023 Sep 20;27:362. doi: 10.1186/s13054-023-04643-y (PMC10512582; doi:10.1186/s13054-023-04643-y)

## Slide 1
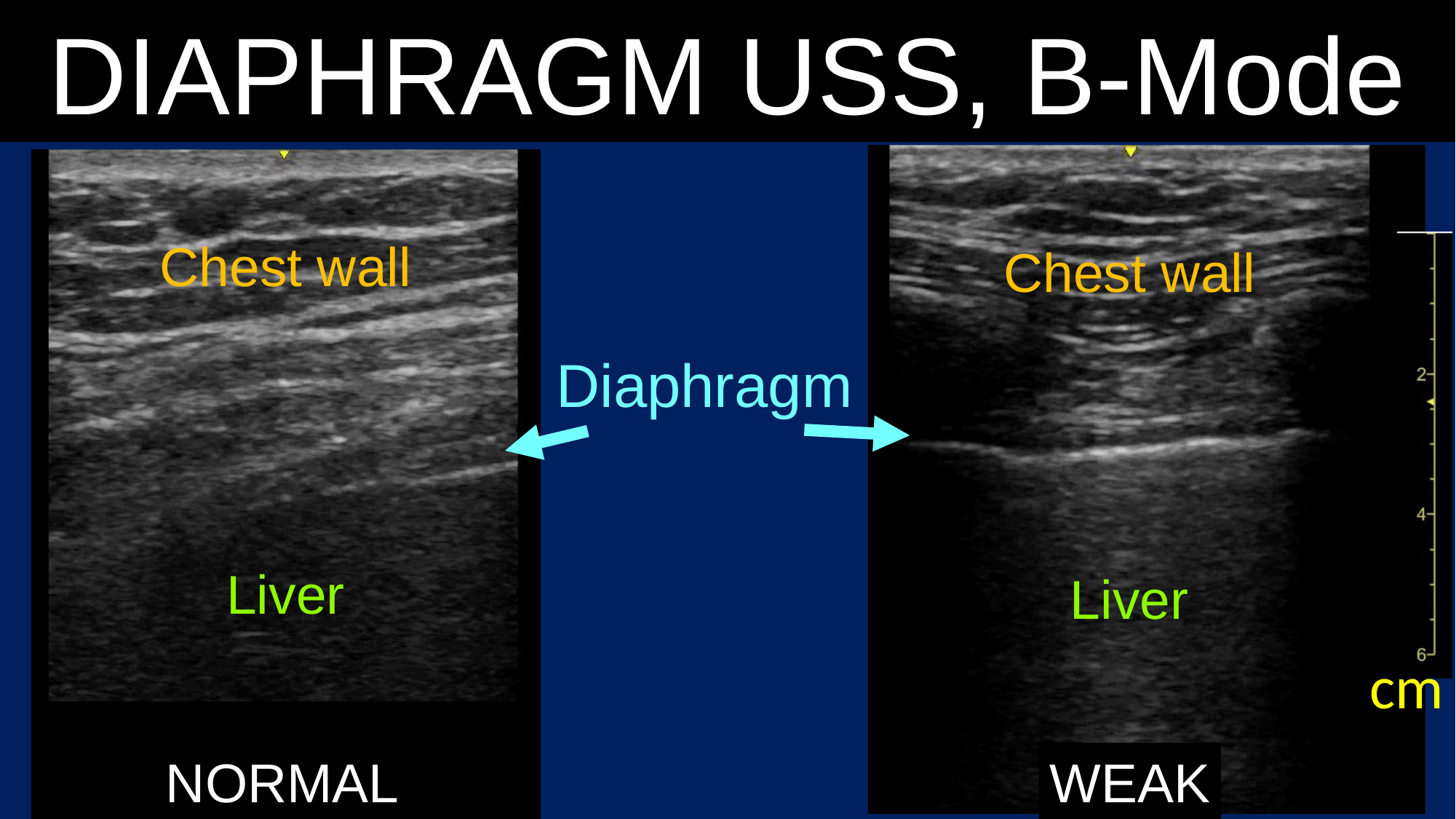

DIAPHRAGM USS, B-Mode
Chest wall
Chest wall
Diaphragm
Liver
Liver
cm
NORMAL
WEAK

Supplement: Supplementary file 3 — Additional file 3: Diaphragmatic ultrasound videos, B-mode [file 13054_2023_4643_MOESM3_ESM.pptx]

## Slide 1
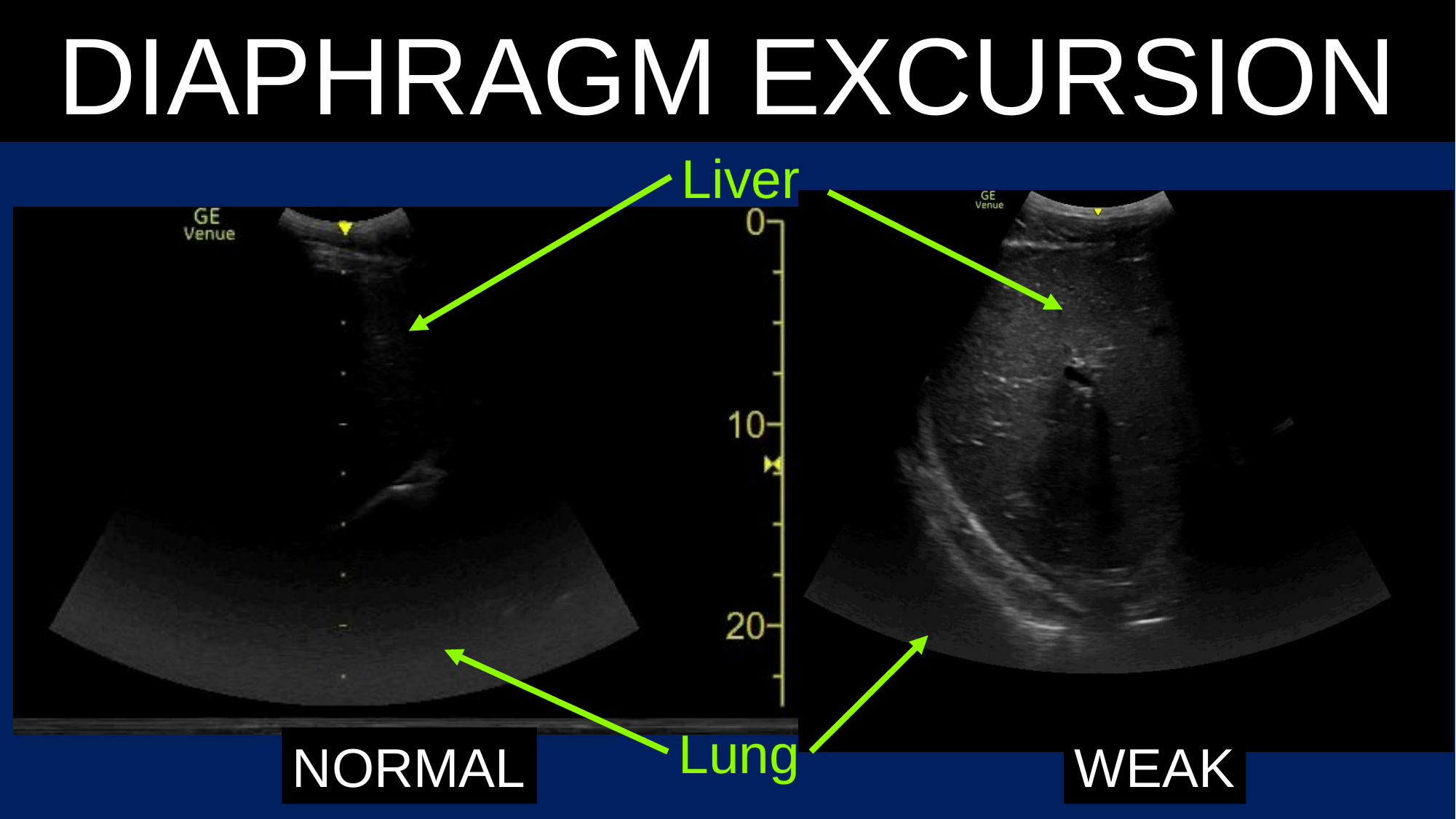

DIAPHRAGM EXCURSION
excursion
Liver
Lung
NORMAL
WEAK

Supplement: Supplementary file 4 — Additional file 4: Diaphragmatic excursion videos, M-mode [file 13054_2023_4643_MOESM4_ESM.pptx]

## Slide 1
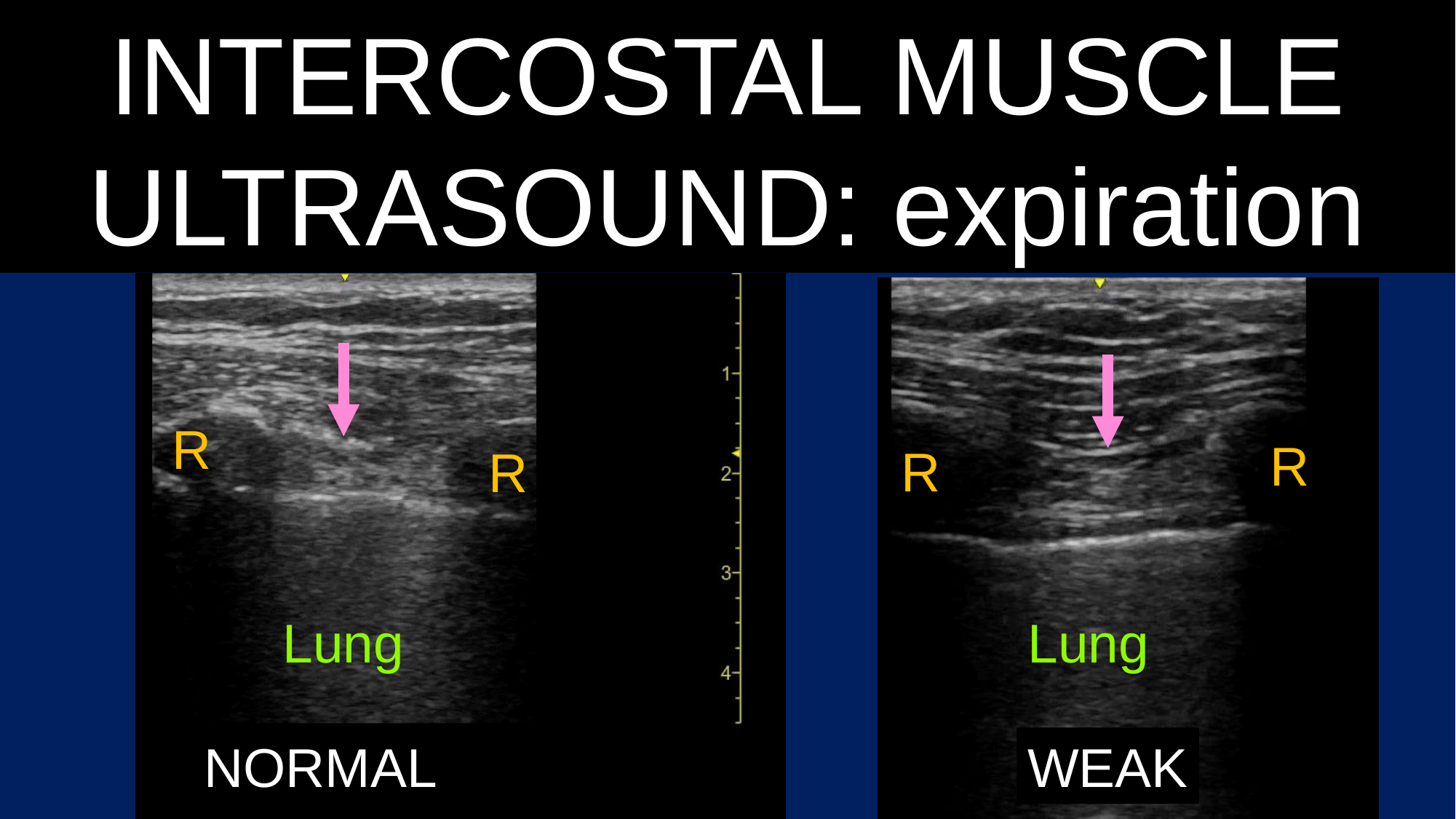

INTERCOSTAL MUSCLE ULTRASOUND: expiration
R
R
R
R
Lung
Lung
NORMAL
WEAK

Supplement: Supplementary file 5 — Additional file 5: Intercostal muscle ultrasound videos [file 13054_2023_4643_MOESM5_ESM.pptx]
