# Supplementary material for: Spinal cord perfusion pressure correlates with breathing function in patients with acute, cervical traumatic spinal cord injuries: an observational study
Source: Crit Care. 2023 Sep 20;27:362. doi: 10.1186/s13054-023-04643-y (PMC10512582; doi:10.1186/s13054-023-04643-y)
Supplement: Supplementary file 6 — Additional file 6: Summary of correlations between individual patient’s breathing function vs SCPP [file 13054_2023_4643_MOESM6_ESM.pdf]

ADDITIONAL FILE 6 – Individual patient correlations of breathing function vs. SCPP

MEASURE OF BREATHING FUNCTION	NO. OF PATIENTS	CORRELATION vs. SCPP				
		ALL PATIENTS COMBINED	INDIVIDUAL PATIENTS (% PATIENTS)			
			↑	↓	∩	→
**EDI-peak**	7	↑	71.4			28.6
**EDI-min**	7	↑	71.4			28.6
**Diaphragm excursion (quiet)**	11	→	27.3			72.7
**Diaphragm excursion (deep)**	11	∩	27.3		63.6	9.1
**Diaphragm thickness (% change quiet)**	11	∩	9.1		81.9	9.1
**Diaphragm thickness (% change deep)**	11	∩	27.3		72.7	
**Upper intercostal thickness (% change)**	11	∩			81.8	18.2
**Middle intercostal thickness (% change)**	11	∩	9.1	9.1	72.7	9.1
**Lower intercostal thickness (% change)**	10	↑	40.0		60.0	
