# Supplementary material for: Spinal cord perfusion pressure correlates with breathing function in patients with acute, cervical traumatic spinal cord injuries: an observational study
Source: Crit Care. 2023 Sep 20;27:362. doi: 10.1186/s13054-023-04643-y (PMC10512582; doi:10.1186/s13054-023-04643-y)
Supplement: Supplementary file 7 — Additional file 7: Plots of relations between individual patient’s breathing function vs SCPP [file 13054_2023_4643_MOESM7_ESM.pdf]

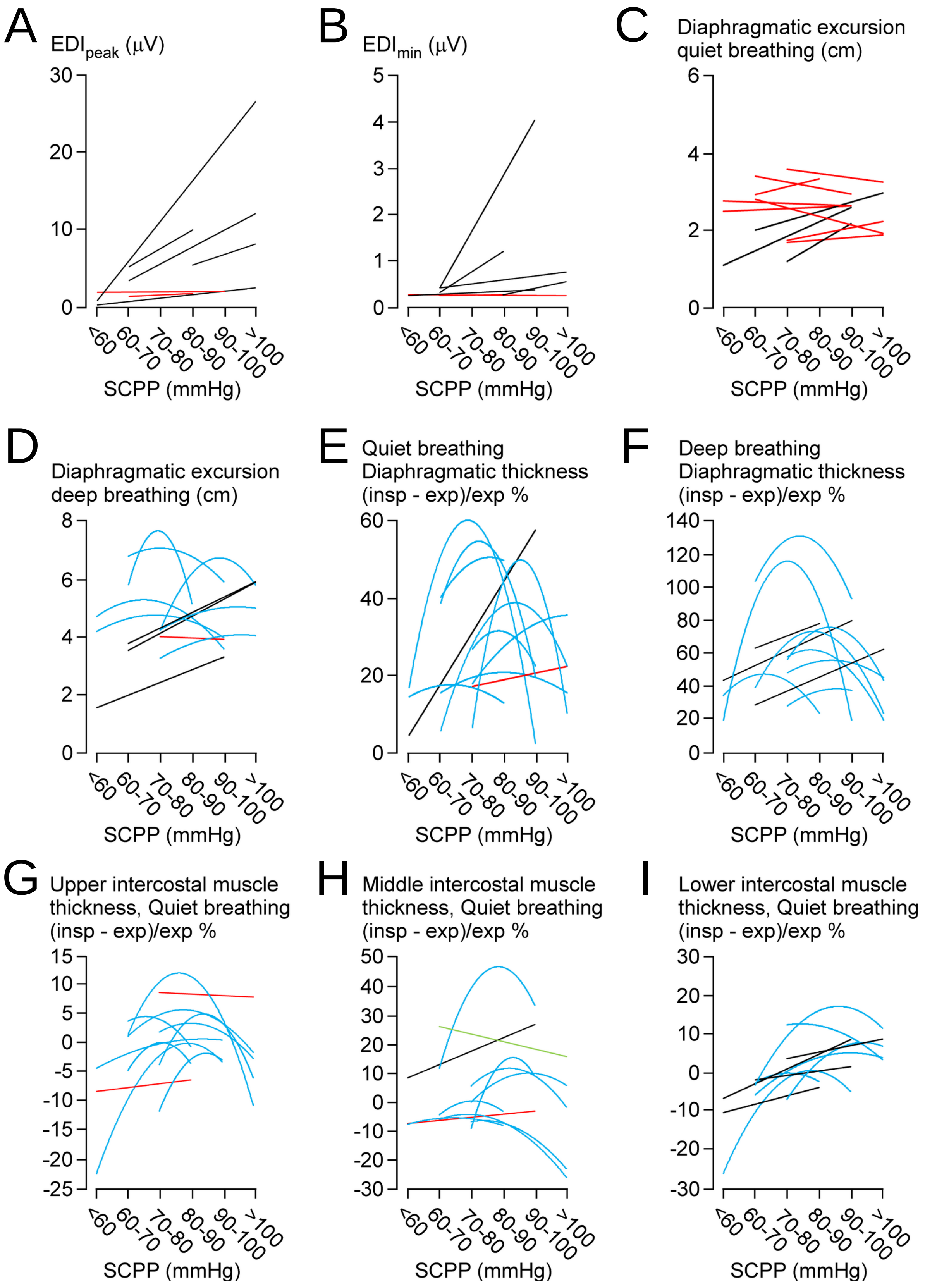

ADDITIONAL FILE 7. Individual patient correlations of breathing function *vs.* SCPP. A. EDI_{peak} **B.** EDI_{min}. Diaphragmatic excursion (cm) during **C.** Quiet breathing, and **D.** Deep breathing. Change in diaphragmatic thickness (inspiration - expiration)/expiration % during **E.** Quiet breathing, and **F.** Deep breathing. Change in muscle thickness (inspiration - expiration)/expiration % during quiet breathing for **G.** Upper intercostal, **H.** Middle intercostal, and **I.** Lower intercostal. Best-fit linear with positive correlation (black), no correlation (red), and negative correlation (green). Best fit quadratic (blue).
