# Supplementary material for: Spinal cord perfusion pressure correlates with breathing function in patients with acute, cervical traumatic spinal cord injuries: an observational study
Source: Crit Care. 2023 Sep 20;27:362. doi: 10.1186/s13054-023-04643-y (PMC10512582; doi:10.1186/s13054-023-04643-y)
Supplement: Supplementary file 8 — Additional file 8: Sample sizes for each graph [file 13054_2023_4643_MOESM8_ESM.pdf]

ADDITIONAL FILE 8. Sample sizes

FIG. 2. Sample sizes.

	2B black	2C black	2B blue	2C blue
<60	8	6	5	5
60 – 70	31	30	8	8
70 – 80	54	52	26	25
80 – 90	55	53	22	23
90 – 100	37	34	3	3
>100	6	5		

FIG. 3. Sample sizes.

	3C black	3D black	3C blue	3D blue
<60	8	7	4	4
60 – 70	22	22	8	6
70 – 80	43	48	28	26
80 – 90	71	68	43	41
90 – 100	31	32	5	16
>100	11	12	9	8
Control	19	20		

FIG. 4. Sample sizes.

	4C black	4D black	4C blue	4D blue
<60	6	6	2	4
60 – 70	14	13	4	8
70 – 80	33	33	15	28
80 – 90	52	52	26	43
90 – 100	20	20	5	16
>100	9	8	9	8
Control	19	20		

FIG. 5. Sample sizes.

	5C black	5D black	5E black	5C blue	5D blue	5E blue
<60	8	8	8	4	4	4
60 – 70	24	24	24	6	8	8
70 – 80	50	50	48	26	28	28
80 – 90	80	80	74	41	44	44
90 – 100	36	36	28	16	18	18
>100	12	12	12	8	8	8
Control	20	19	8			

ADDITIONAL FILE 2. Sample sizes.

	$p_{sct}O_2$	Glucose	LPR
<60	28	19	24
60 – 70	119	148	169
70 – 80	283	429	424
80 – 90	276	437	413
90 – 100	125	156	175
>100	16	29	25

ADDITIONAL FILE 7. Correlation r^2

A	B	C	D	E	F	G	H	I
0.02	0.09	0.01	0.00	0.07	0.63	0.05	0.62	0.58
0.03	0.17	0.02	0.33	0.41	0.63	0.26	0.10	0.54
0.33	0.37	0.02	0.37	0.46	0.74	0.39	0.40	0.31
0.79	0.77	0.05	0.40	0.61	0.76	0.66	1.00	0.32
0.85	0.90	0.07	0.80	0.70	0.92	0.88	1.00	0.37
0.89	0.99	0.07	0.89	0.88	0.95	0.88	0.72	1.00
0.90	1.00	0.11	0.97	1.00	0.98	1.00	0.90	0.96
		0.11	0.97	1.00	0.99	1.00	1.00	0.93
		0.44	1.00	1.00	1.00	1.00	1.00	0.95
		0.78	1.00	1.00	1.00	1.00	0.95	1.00
		0.82	1.00	1.00	1.00	1.00	1.00	
